# Can we screen for heart disease in children at public health centres? A multicentre observational study of screening for heart disease with a risk of sudden death in children

**DOI:** 10.1007/s00431-024-05489-4

**Published:** 2024-03-08

**Authors:** Paula Greciano Calero, Silvia Escribá Bori, Juan Antonio Costa Orvay, Nina González Pons, María del Carmen Martín Pérez, Dolores Cardona Alfonseca, Cristina Nogales Velázquez, Sergi Verd Vallespir, Alicia Esther Tur Salom, Antonella Chiandetti, Marcos Navarro Noguera, Anna Grau Blanch, María Magdalena Rotger Genestar, Marianna Mambié Meléndez, Mercedes Fernández Hidalgo, Juana María Seguí Llinas, Laura Martorell Bon, Patricia Arestuche Aguilar, Beatriz Garrido Conde, María del Valle Sánchez Grao, Katia Sarraff Trujillo, Antoni Muntaner Alonso, Catalina Grimalt Ferragut, Andrea Soriano Marco, Viviana Gómez Rojas, Juan Pol Serra

**Affiliations:** 1 Children’s Heart Unit, Paediatric Department, Son Espases University Hospital, Palma, Balearic Islands, Spain; 2 Children’s Heart Unit, Paediatric Department, Can Misses Hospital, Ibiza, Balearic Islands, Spain; 3 Children’s Heart Unit, Paediatric Department, Mateu Orfila General Hospital, Mahon, Balearic Islands, Spain; 4 Son Ferriol Public Health Centre, Son Ferriol, Balearic Islands, Spain; 5 Son Serra-La Vileta Public Health Centre, Palma, Balearic Islands, Spain; 6 Vila Public Health Centre, Ibiza, Balearic Islands, Spain; 7 Es Mercadal Public Health Centre, Es Mercadal, Balearic Islands, Spain; 8 Es Blanquer Public Health Centre, Inca, Balearic Islands, Spain; 9 S’Escorxador Public Health Centre, Palma, Balearic Islands, Spain; 10 Muntanya Public Health Centre, Son Ramonell Nou, Balearic Islands, Spain

**Keywords:** Sudden death, Heart disease, Screening, Children, Primary care, Electrocardiogram

## Abstract

Sudden cardiac death in children is a rare event, but of great social significance. Generally, it is related to heart disease with a risk of sudden cardiac death (SCD), which may occur with cardiovascular symptoms and/or electrocardiographic markers; thus, a primary care paediatrician (PCP) could detect them. Therefore, we proposed a study that assesses how to put into practice and conduct a cardiovascular assessment within the routine healthy-child check-ups at six and twelve years of age; that reflects cardiovascular signs and symptoms, as well as the electrocardiographic alterations that children with a risk of SCD in the selected population present; and that assesses the PCP’s skill at electrocardiogram (ECG) interpretation. In collaboration with PCPs, primary care nurses, and paediatric cardiologists, an observational, descriptive, multicentre, cross-sectional study was carried out in the Balearic Islands (Spain), from April 2021 to January 2022, inclusive. The PCPs gathered patient data through forms (medical record, electrocardiogram, and physical examination) and sent them to the investigator, together with the informed consent document and electrocardiogram. The investigator passed the electrocardiogram on to the paediatric cardiologists for reading, in an identical form to those the paediatricians had filled in. The variables were collected, and a descriptive analysis performed. Three paediatric cardiologists, twelve PCPs, and nine nurses from seven public health centres took part. They collected the data from 641 patients, but 233 patients did not participate (in 81.11% due to the PCP’s workload). Therefore, the study coverage was around 64%, representing the quotient of the total number of patients who participated, divided by the total number of patients who were eligible for the study. We detected 30 patients with electrocardiographic alterations compatible with SCD risk. Nine of these had been examined by a paediatric cardiologist at some time (functional murmur in 8/9), five had reported shortness of breath with exercise, and four had reported a family history of sudden death. The physical examination of all the patients whose ECG was compatible with a risk of SCD was normal. Upon analysing to what extent the ECG results of the PCP and the paediatric cardiologist agreed, the percentage of agreement in the final interpretation (normal/altered) was 91.9%, while Cohen’s kappa coefficient was 31.2% (CI 95%: 13.8–48.6%). The sensitivity of the ECG interpretation by the PCP to detect an ECG compatible with a risk of SCD was 29% and the positive predictive value 45%.

*     Conclusions*: This study lays the foundations for future SCD risk screening in children, performed by PCPs. However, previously, it would be important to optimise their training in reading and interpreting paediatric ECGs.

**Table:** 

**What is Known:** • *In Spain at present, there is a programme in place to detect heart disease with a risk of sudden death *[1]*, but it targets only children who are starting on or are doing a physical activity as a federated sport. Implementing such screening programmes has proven effective in several countries *[2]*. However, several studies showed that the incidence of sudden cardiac death is no higher in children competing in sport activities than in those who do not do any sport *[3]*. This poses an ethical conflict, because at present, children who do not do any federated sport are excluded from screening. According to the revised literature, so far, only in two studies did they screen the child population at schools, and in both, they successfully detected patients with heart disease associated to the risk of sudden death *[4, 5]*. We have found no studies where the screening of these features was included within the routine healthy-child check-ups by primary care paediatricians.*	
**What is New:** • *We did not know whether—in our setting, at present—the primary care paediatrician could perform a screening method within the routine healthy-child check-ups, in order to detect presumably healthy children at risk of sudden cardiac death, as they present one of the SCD risks. In this regard, we proposed our project: to assess how to put into practice and conduct a cardiovascular assessment via SCD risk screening in the healthy child population by primary care paediatricians and appraise primary care paediatricians’ skills in identifying the electrocardiographic alterations associated with SCD risk. The ultimate intention of this pilot study was to make it possible, in the future, to design and justify a study aimed at universalising cardiovascular screening and achieving a long-term decrease in sudden cardiac death events in children.*

**What is Known:**

• *In Spain at present, there is a programme in place to detect heart disease with a risk of sudden death *[1]*, but it targets only children who are starting on or are doing a physical activity as a federated sport. Implementing such screening programmes has proven effective in several countries *[2]*. However, several studies showed that the incidence of sudden cardiac death is no higher in children competing in sport activities than in those who do not do any sport *[3]*. This poses an ethical conflict, because at present, children who do not do any federated sport are excluded from screening. According to the revised literature, so far, only in two studies did they screen the child population at schools, and in both, they successfully detected patients with heart disease associated to the risk of sudden death *[4, 5]*. We have found no studies where the screening of these features was included within the routine healthy-child check-ups by primary care paediatricians.*

**What is New:**

• *We did not know whether—in our setting, at present—the primary care paediatrician could perform a screening method within the routine healthy-child check-ups, in order to detect presumably healthy children at risk of sudden cardiac death, as they present one of the SCD risks. In this regard, we proposed our project: to assess how to put into practice and conduct a cardiovascular assessment via SCD risk screening in the healthy child population by primary care paediatricians and appraise primary care paediatricians’ skills in identifying the electrocardiographic alterations associated with SCD risk. The ultimate intention of this pilot study was to make it possible, in the future, to design and justify a study aimed at universalising cardiovascular screening and achieving a long-term decrease in sudden cardiac death events in children.*

## Introduction

Sudden cardiac death in children is a rare event (2.28/100000 people-year) [6]. Nonetheless, occurring in this age group, it has a significant social impact [7]. At the root of the episodes of sudden cardiac death are mainly diseases such as hypertrophic cardiomyopathy, dilated cardiomyopathy, arrhythmogenic right ventricular dysplasia, long and short QT syndrome, Wolff-Parkinson-White syndrome, Brugada syndrome, and catecholaminergic polymorphic ventricular tachycardia [6, 8] (hence, we refer to these conditions as heart disease with a risk of sudden death or SCD risk). During their clinical course, some of the aforementioned conditions present with cardiovascular symptomatology and/or show electrocardiographic markers of risk of sudden cardiac death [9, 10]. Further, patients with one of these conditions sometimes have a family history of heart disease or sudden death.

Most patients undergoing an episode of sudden death showed very unspecific earlier symptoms, and a significant percentage even present with sudden death as the first symptom [10]. Not surprisingly, previous series have shown that the data of the medical record and the physical examination in isolation—as the *American Heart Association* promotes—barely have any sensitivity, varying around 20% for anamnesis and 10% for physical examination, with a specificity of between 70 and 90% for both. This fact reveals that there is a need for a method that complements anamnesis and physical examination. In this regard, the ECG in paediatrics is a simple, quick, inexpensive, painless, and accessible test, in both primary and hospital care, and its demonstrated sensitivity and specificity are greater than 90% [11, 12]. In conclusion, the factors associated with SCD risk could be detected by a primary care paediatrician by means of the medical record, physical examination, and ECG reading during a routine consultation [13]. Knowing these factors, more effective ways of screening for these conditions could be established.

In Spain, at present, there is a programme in place to detect heart disease with a risk of sudden death [1], but it targets only children who are starting on or are doing a physical activity as a federated sport [1, 14]. Implementing screening programmes has proven effective in several countries [2]. However, several studies showed that the incidence of sudden cardiac death is no higher in children competing in sport activities than in those who do not do any sport [3, 15], which poses an ethical conflict, because at present, children who do not do any federated sport are excluded from screening. According to the revised literature, so far, only in two studies did they screen the child population at schools, and in both, they successfully detected patients with heart disease associated to the risk of sudden death [4, 5]. We have found no studies where the screening of these features was included within the routine healthy-child check-ups by the primary care paediatricians.

We do not know whether—in our setting, at present—the primary care paediatrician could perform a screening method within the routine healthy-child check-ups, in order to detect presumably healthy children at risk of sudden cardiac death, as they present one of the risks of SCD. In this respect, we proposed a project that aimed to: assess how to put into practice and conduct cardiovascular assessment within the routine healthy-child check-ups; by reflecting cardiovascular symptoms and signs, as well as the electrocardiographic alterations present in children with a risk of SCD in the selected population; and, finally, assessing the primary care paediatricians’ skill at interpreting an electrocardiogram (ECG) and detecting the electrocardiographical signs of SCD risk. The study was named ECIAP (*Evaluación Cardiovascular Infantil en Atención Primaria*) Study: Cardiovascular Assessment in Children in Primary Care.

## Materials and methods

This multicentre study was conducted in the Autonomous Community of the Balearic Islands, Spain. Its development was coordinated by one principal investigator and the Children’s Heart Units of three hospitals (Son Espases University Hospital, Can Misses Hospital, and Mateu Orfila General Hospital). Paediatricians and nurses in seven public health centres altogether collaborated in the collection of data; prior to the initiation of the study, they had received instructions to select and include patients in a correct manner.

The study design was observational, descriptive, and cross-sectional. The study population selected were healthy children without any known heart disease who went to the participating public health centres for their routine healthy-child check-ups at the age of 6 and 12 years. The data of as many patients as possible were collected during the pre-established duration of the project (April 2021 to January 2022, inclusive); therefore, no particular sample size was established. The inclusion criteria were being a child in the selected population, in addition to: their father, mother, or guardian having granted their informed consent; having filled in the data collection forms; and being 5.5 to 7 years of age, in the case of the 6-year check-up, and 11.5 to 13 years of age, in the case of the 12-year check-up. The only exclusion criteria established were presenting a patient history record of heart disease, or having been categorised as a complex chronic patient.

**Fig. 1:**
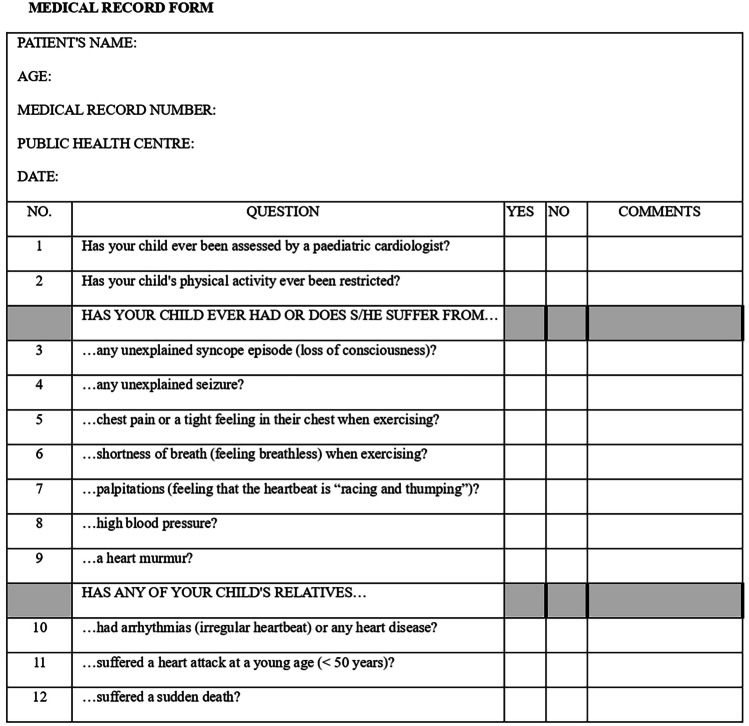
Medical record form

**Fig. 2:**
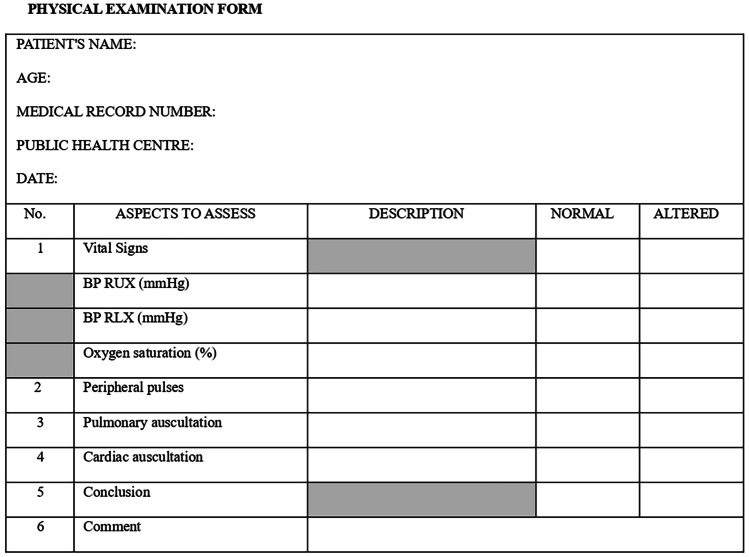
Physical examination form

**Fig. 3.**
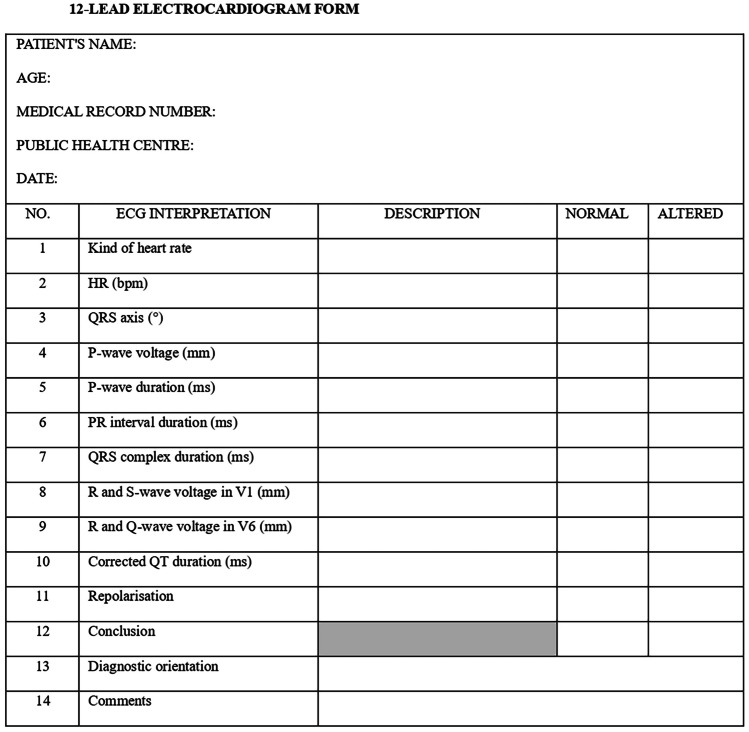
12-Lead electrocardiogram form

The paediatricians in each public health centre obtained the data through three forms (Figs. 1, 2, and 3). They filled in the ECG form from the reading of a 12-lead ECG, made at the public health centre by the nursing staff. Once completed, they sent this together with the informed consent document and the printed ECG to the principal investigator who safeguarded the information. Then, the printed ECG was passed on to the participating paediatric cardiologists, in order to fill in another ECG interpretation form (Fig. 3). The paediatric cardiologists did not know how the public health centre paediatricians had interpreted the ECG or any of the other information provided in the forms. They only knew the age of the patient whose ECG they were to interpret. Subsequently, all the data were collected in an Excel file; only the principal investigator and the statistical analysis staff who carried out a descriptive analysis had access. The correct ECG interpretation was determined to be the one made by the participating paediatric cardiologists.

A descriptive analysis was carried out in order to define the group to be studied; categorical variables were expressed in absolute and relative frequencies (percentages) and numerical variables as the medians and interquartile range. The comparisons between two groups were contrasted by using the Mann–Whitney *U* test for numerical variables, and the Chi-squared or Fisher’s exact test for categorical variables; a *p* value < 0.05 was taken as the indicator of a significant difference. The sensitivity, specificity, PPV, and NPV indicators of the paediatricians' criteria with regard to the cardiology physicians’ criteria were calculated as the gold standard, while the study of the degree of agreement was assessed using the Kappa Index. The Methodological and Statistical Support Platform of the Health Research Institute of the Balearic Islands (IdISBa) developed our data analysis; the statistical software used was IBM SPSS v. 26.

## Results

All in all, twelve primary care paediatricians and nine nurses from seven health centres on the Balearic Islands participated, as well as three paediatric cardiologists. Data were collected from 641 patients in all, 408 of whom finally participated in the study. Therefore, the study coverage was 64%, representing the quotient of the total number of patients who participated, divided by the total number of patients who were eligible for the study. Of the 233 patients who did not participate, in 81.11% of cases, the reason was that the paediatrician’s workload prevented them from offering the study to the patient’s father/mother/guardian, giving the appropriate information, and/or completing the forms; whereas in 16.73% of patients, the cause was that the father, mother, or guardian did not grant their consent. Five patients were excluded from the study, as they finally did not fulfil the inclusion criteria (they had some kind of heart disease).

Regarding the characteristics of the 408 patients who participated, gender distribution was homogeneous, with 50% female patients. Just over half (55.14%) were patients who went for the 6-year check-up. In 21% of the patients who participated in the study, part of it had to be postponed because it could not be performed within the time set aside for the routine check-up.

In the descriptive analysis, we found variability in the coverage depending on the participating health centre, with a coverage range from 87% at the Son Serra-La Vileta Health Centre, to 23% at the Muntanya Health Centre (Table 1).

**Table 1:** Descriptive analysis

Public health centre	Patients (%)	No paediatrician available (%)	No authorisation (%)	Excluded (%)	Participants (%)
Son Serra-La Vileta	159 (24.8%)	10 (6.3%)	11 (6.9%)	0	138 (86.8%)
Es Blanquer	95 (14.82%)	27 (28.4%)	16 (16.8%)	0	52 (54.7%)
Muntanya	60 (9.36%)	46 (76.7%)	0	0	14 (23.3%)
S´Escorxador	92 (14.35%)	46 (50%)	6 (6.5%)	0	40 (43.5%)
Son Ferriol	96 (14.97%)	25 (26%)	2 (2.1%)	0	69 (71.9%)
Vila	78 (12.16%)	21 (26.9%)	4 (5.1%)	2 (2.6%)	51 (65.4%)
Es Mercadal	61 (9.51%)	14 (23%)	0	3 (4.9%)	44 (72.1%)
Total	641 (100%)	189 (29.5%)	39 (6.1%)	5 (0.8%)	408 (63.7%)

The study detected a total of 30 electrocardiographic alterations that could be related to a heart disease with risk of sudden death:

- 13 patients with borderline QT interval
- 4 patients with long QT interval
- 4 patients with left heart axis
- 4 patients with abnormal repolarisation
- 3 patients with signs of left ventricular enlargement
- 1 patient with short QT interval
- 1 patient with pre-excitation syndrome

Further, other electrocardiographic alterations, unrelated to SCD risk, were detected:

- 1 patient with first grade atrioventricular block
- 6 patients with low atrial rhythm
- 1 patient with notched P morphology
- 2 patients with a larger than normal S-wave amplitude in V1
- 4 patients with a low alternans of the sinus and atrial rhythm

Twelve of the 30 patients whose ECGs were considered pathological, as they presented signs compatible with a risk of SCD, reported some family history of heart disease (in no case of SCD risk) in the medical record form; nine reported that at some time, they had been examined by a paediatric cardiologist (none of them had actively followed-up at the time of the study); eight reported that they had been told about a heart murmur during childhood; five reported shortness of breath during exercise; and four reported a family history of sudden death. No case—in either the group whose ECG was compatible with SCD risk or the group whose ECG was not compatible with SCD risk—was statistically and clinically significant in any of the items regarding the medical record or the physical examination. All the patients whose ECG was compatible with SCD risk had a normal physical examination based on their physical examination form. A pathological ECG was found in 3.11% of the patients who went to the 6-year check-up and in 12.56% of the patients who went to the 12-year check-up.

Upon analysing to what extent, the ECG results of the primary care paediatrician and the paediatric cardiologist agreed, the percentage of agreement in the final interpretation (normal/altered) was 91.9%. However, when analysing to what extent the data agreed with Cohen’s kappa coefficient, the result revealed 31.2% (CI 95%: 13.8–48.6%). The sensitivity of the ECG interpretation by the primary care paediatrician to detect an ECG compatible with SCD risk was 29% and the positive predictive value 45%.

## Discussion

The study was conducted in collaboration with paediatricians and nurses from seven public health centres in the Balearic Islands. It is worth pointing out that collaboration was voluntary; consequently, we must take into account the fact that there is a probability that the involvement of these professionals might not be extrapolated to others in the same setting, which would represent a limitation to this study. Some of the participating professionals at the health centres were not trained paediatricians, but rather Family and Community Medicine physicians who had been practising primary care paediatrics for years. We consider their participation as positive because it actually represents the situation when applying the screening, since in the Balearic Islands, as well as in many other Spanish autonomous communities, these physicians are the professionals who practise paediatrics in primary care in some cases.

The coverage of the screening in the selected population was 64%, which suggests that improvements in the patient care system are needed in order to apply this screening in public health centres. After completion of this study and in view of the results, we concluded that it would be possible to put into practice and conduct SCD risk screening through cardiovascular assessment within the routine healthy-child check-ups, but it would require staff improvements in the public health centres and/or increasing the time for check-ups assigned to every patient. In part, the variability between paediatricians and health centres may be due to the voluntary nature of the study and each paediatrician’s subjective perception of the time needed to carry it out. An interesting fact to evidence is that in 21% of the patients included in the study, some of the forms were filled in, or the ECGs done, later, and compared to the others, they were included the same as patients without differences. There could be some discussion as to whether those patients should finally be considered participants or not.

Altogether, 30 alterations of the ECG compatible with a risk of SCD were detected, representing 7.35% of patients. The criteria to consider them as alterations were based on the paediatric cardiologists’ ECG form. The inclusion of patients with borderline QT interval in this category is debatable [16]. Although the study was not aimed at detecting other electrocardiographic alterations than those indicating a risk of SCD, it was interesting to learn what other alterations were detected. After completing the study, the paediatricians in the health centres were informed of the alterations detected in their patients so that they could be referred to the corresponding Children’s Heart Unit. It would be of significant interest to investigate and analyse the ultimate diagnoses of those patients referred to cardiology due to the identification of a pathological electrocardiogram in this study. This knowledge could offer valuable insights into the effectiveness of early detection of cardiovascular abnormalities in the paediatric population. The percentage of patients who went to the 12-year check-up and showed an ECG compatible with SCD risk was four times higher than that of patients who went to the 6-year check-up (12.56% and 3.11%, respectively). The information obtained by this study, as well as the current knowledge of the evolving process of SCD risk, lead us to think that if SCD risk screening is implemented in primary care, it might be more effective if carried out in the 12-year check-up than in the 6-year check-up, but there are no studies supporting the screening at a specific age, and due to its characteristics, this study would also not serve to make a solid recommendation in this regard.

The results show that no case—either in the group whose ECG was compatible with SCD risk or the group whose ECG was not compatible with SCD risk—was statistically and clinically significant in any of the items assessed by the medical record or the physical examination. This information could be interesting for establishing what information from the medical record and physical examination was relevant for setting a screening of those characteristics. In addition, it would be important to specify what family cases and what degree of relationship were relevant to be included in item ten of the medical record form, as a large variability of conditions was observed in the answers, and their significance is debatable, so no further studies about this data were conducted.

As we said, in this study, it has been intriguing to observe how the responses from patients and their families to these three questions had little value. In some cases, health or cardiac problems unrelated to sudden death were included in the family history, and information about relatives who were not significantly related to the patient was provided. It would be interesting to guide these questions in a way that specifies which background information is relevant. The family history can be a real risk factor for SCD [10]. The most well-known questionnaire on this topic was recommended by the American Heart Association [17], consisting of 14 questions, including three related to family history. We believe that these questions are more targeted and specify some key entities, which may facilitate responses from patients and families. In future studies, the modification of this medical history form should be considered. Additionally, upon reviewing the literature, we have found that in many cases, positive responses to aspects of the medical history in this well-known 14-question questionnaire used by the American Heart Association (and on which our Medical Record Form is based) significantly decrease after a physician's review of the responses [18]. It is interesting to note that in our study, paediatricians were not required to review the responses of patients and families, so the validity of the answers to this questionnaire, as we have already mentioned, is debatable. For future studies, it would be interesting to have the PCP jointly review the questionnaire with the family after its completion to adjust the responses and ensure they are truly clinically concerning for cardiac disease.

The analysis of the agreement of the ECG reading between paediatrician and paediatric cardiologist was positive, with a high degree of agreement (91.9%) in the final interpretation of the ECG regarding whether it was normal or altered. However, when correcting with Cohen’s kappa coefficient to what extent the data agreed, taking into account the random probability of agreement, the result showed a much lower [31.2% (CI 95%: 13.8–48.6%)], moderate agreement between paediatrician and paediatric cardiologist. Cohen’s kappa coefficient is affected by prevalence; thus, the explanation for the quantitative decrease in degree of agreement would seem to be the substantially high prevalence of ECGs categorised as normal (92.64%), as opposed to pathological. The results show that the sensitivity of the ECG interpretation by the primary care paediatrician to detect an ECG compatible with a risk of SCD was 29% and the positive predictive value 45%. Both values are far from optimal figures for a screening method. This implies that currently, the paediatricians in public health centres need both specific training in systematic electrocardiogram-reading and to improve their sensitivity for them to be able to perform SCD risk screening. Other studies also have shown that modest agreement in ECG interpretation would limit the application of ECG screening [19–24].

In this regard, an important reflection should be carried out. It is true that the sample of PCP is very limited, and the data cannot be generalized to the entire existing PCP staff. It would be, therefore, very interesting to evaluate the electrocardiogram interpretation capability of the entire PCP staff to establish action plans based on the results. In this respect, the Primary Care Management of the Balearic Islands has been informed of the results of this study, and we are aware that a study is being launched to assess the ability of Balearic Islands’ PCPs to interpret electrocardiograms. Based on the overall results, this need or deficiency should be addressed.

It is likely that the use of electrocardiograms in the PCP’s office is not a daily occurrence. Tools or techniques that are not used frequently but are of great importance in clinical practice, such as cardiopulmonary resuscitation, require periodic refreshers [25–27]. Therefore, it would be interesting to propose the creation of refresher courses taught by paediatric cardiologists for the interpretation of electrocardiograms, similar to those proposed for cardiopulmonary resuscitation. The optimal frequency should also be studied. At the same time, it would be interesting to refer to the root of the training of paediatricians and assess the electrocardiogram interpretation capability acquired by paediatric residents, especially focusing on those who do not choose paediatric cardiology as a subspecialty during their training years. If it is not optimal, a significant training deficiency would be evident. This issue has already been evidenced in some articles found in the bibliography [28, 29], which should be addressed by the teaching units of hospitals. Basic training in this aspect should be ensured for all paediatric residents regardless of the paediatric field they reach [30].

There are aspects that might be interesting and have not been assessed in this study. For instance, neither the percentage of child population going to private centres for their routine check-ups, nor the impact of putting into practice and conducting the study at children’s heart clinics was taken into account. We do not know how many more referrals to children's heart specialists were made, or what additional workload the screening of those characteristics means for professionals. Furthermore, the study does not assess the economical cost associated with the screening or the subsequent referrals made. However, it is an important issue that should be thoroughly evaluated in future screening programs. Nor has it been taken into account which patients among those who participated engaged in competitive sports and which did not. This aspect is significant because currently, SCD screening is conducted only on federated patients in most countries, despite data indicating that these patients do not have a higher risk [3, 31]. It would have been interesting to determine this condition among patients due to the phase in which the screening currently stands.

It is important to note that there is still significant controversy surrounding the appropriateness of screening for cardiac conditions with the risk of sudden death. There are both supporters and detractors, mainly because there are unresolved and unsecured aspects of the criteria for implementing a screening program regarding entities that can cause sudden death [13, 32, 33]. One of these criteria is that there must be a simple, safe, precise, and validated screening test. Baseline electrocardiography, even when supported by medical history and physical examination, may not detect some of these cardiac conditions, such as hypertrophic cardiomyopathy in early stages in children, arrhythmogenic right ventricular cardiomyopathy, anomalous origin of a coronary artery, and catecholaminergic polymorphic ventricular tachycardia. The sensitivity of screening ECGs for the various channelopathies, preexcitation syndromes and cardiomyopathies can be difficult to stablish with precision, because disease severity within populations affects the prevalence and extent abnormalities on the ECG and because phenotypic expression of these disorders is heterogeneous [34–37].

This study lays the foundations for another one with a larger sample that would establish the effectiveness of screening heart disease with a risk of sudden death in childhood by primary care paediatricians. It can serve as a model to design and justify a pilot project of screening, with the aim of making SCD risk screening in children universal and, as such, reduce the events of sudden cardiac death in the long term. One significant prerequisite is boosting primary care paediatricians’ training for reading and interpreting paediatric ECGs. This study prompted a training project in the Balearic Islands, aimed at primary care paediatricians, with the objective of subsequently starting a pilot project of SCD risk screening.

## Data Availability

No datasets were generated or analysed during the current study.

## Abbreviations

BP RUX: Blood pressure measured in right upper extremity
BP RLX: Blood pressure measured in right lower extremity
CI: Confidence interval
ECG: Electrocardiogram
NPV: Negative predictive value
PCP: Primary care paediatrician
PPV: Positive predictive value
SCD risk: Risk of sudden cardiac death
